# A Case Report and Review of the Literature: Infectious Aneurysm Formation in the Pulmonary Arteries—A Rare but Perilous Sequela of Persisting Infection With *Klebsiella pneumoniae*

**DOI:** 10.3389/fmicb.2022.893737

**Published:** 2022-05-17

**Authors:** Jannik Ruwisch, Bettina Fischer, Lea Häbel, Florian Laenger, Benjamin-Alexander Bollmann

**Affiliations:** ^1^Clinic for Respiratory Medicine, Hannover Medical School, Hannover, Germany; ^2^Biomedical Research in End Stage and Obstructive Lung Disease, German Center for Lung Research, Hannover, Germany; ^3^Clinic for Cardiology and Angiology, Hannover Medical School, Hannover, Germany; ^4^Department of Pathology, Hannover Medical School, Hannover, Germany

**Keywords:** infectious aneurysm, haemoptoe, urosepsis, *Klebsiella pneumoniae*, pulmonary artery

## Abstract

Septic aneurysms of the pulmonary artery are rare conditions, with few cases having been reported worldwide. They are assumed to result from septic emboli that cause a local inflammatory reaction of the arterial wall, ultimately leading to degenerative changes. We report the case of a 63-year-old female patient presenting with *Klebsiella pneumoniae* urosepsis and first diagnosis of diabetes mellitus, who developed a life-threatening infectious pulmonary artery aneurysm secondary to bacteremia with *Klebsiella pneumoniae*. The patient required a lobectomy due to pulmonary hemorrhage. We review the clinical hallmarks of *Klebsiella pneumoniae* related septic pulmonary embolic disease and summarize currently known risk factors for the development of infectious aneurysmatic disease including diabetes mellitus and other states of immunosuppression. The featured case aims to increase the awareness for this seldom but life-threatening complication of infectious diseases such as *Klebsiella pneumoniae* urosepsis.

## Introduction

The term pulmonary infectious (former mycotic) aneurysm describes an infection-related ectasia of the pulmonary artery due to prolonged bacterial destruction of the arterial wall (Majeed and Ahmad, 2021). The term “mycotic” is actually a historical misnomer, first introduced by Sir William Osler in 1885 (Osler, 1885), as mostly bacterial and not mycotic pathogens trigger infectious aneurysms. Aneurysm formation of the pulmonary arteries is a rare event with a high fatality rate (Bartter et al., 1988). It may result from bacteremia or septic emboli that cause degenerative changes in the arterial wall (Brown et al., 1984; Macbeth et al., 1984; Qureshi et al., 1999; Oderich et al., 2001). In this regard, risk factors and biomarkers for aneurysm formation under persistent infection despite systemic antibiotic treatment are missing, leaving the patient at risk for undetected disease progression and critical pulmonary complications. The following case highlights these mentioned difficulties and gives brief overview of the literature on *Klebsiella pneumoniae* (*KP*) related septic pulmonary disease and its role in aneurysm formation.

## Case-Report

**Figure 1:**
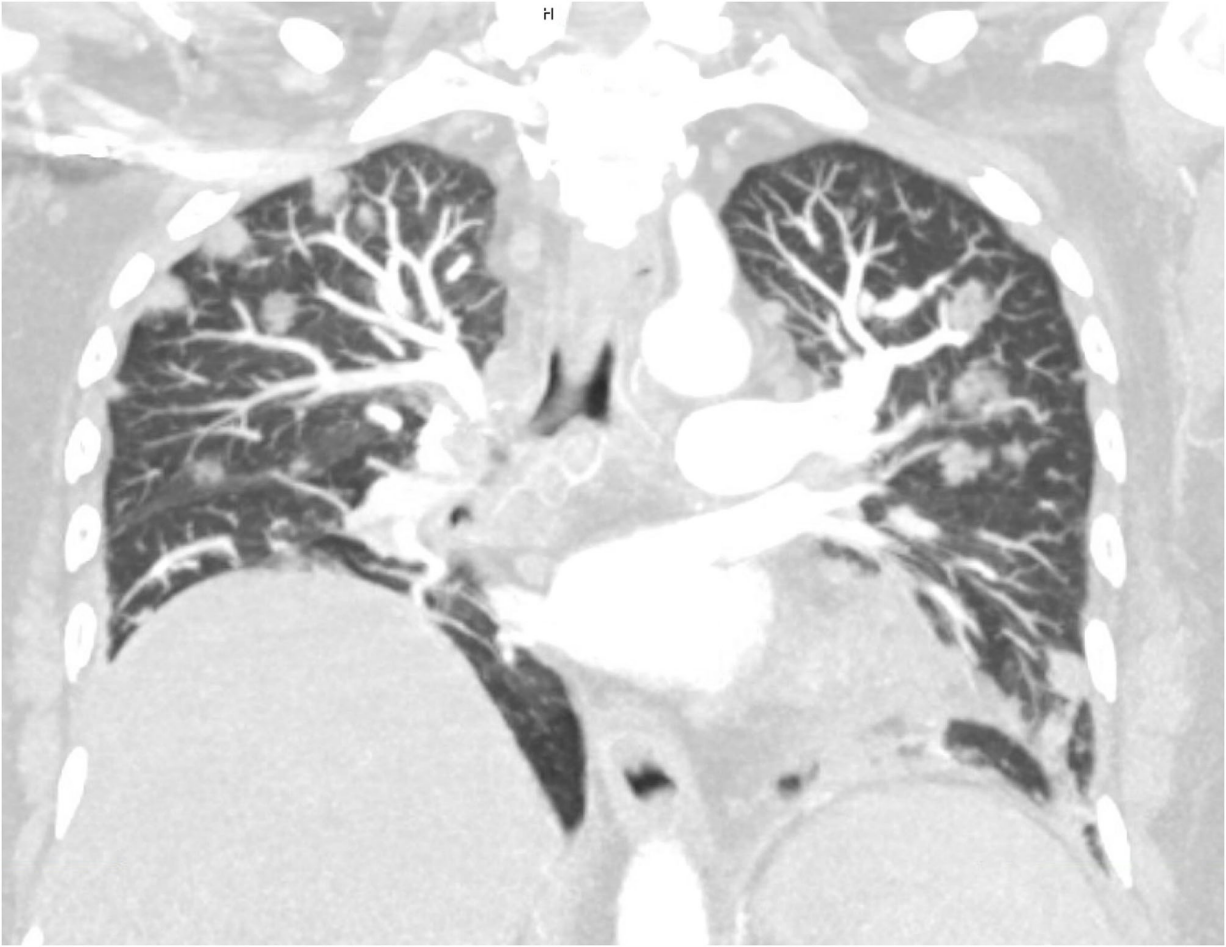
Maximal intensity projection (MIP) of multi-planar reconstruction computed tomographic angiography at admission day. Multiple septic embolic lesions appearing as nodules are present predominantly in the subpleural as well as peribronchovascular area, while close topographic proximity to the branching pulmonary arteries is noted.

**Figure 2:**
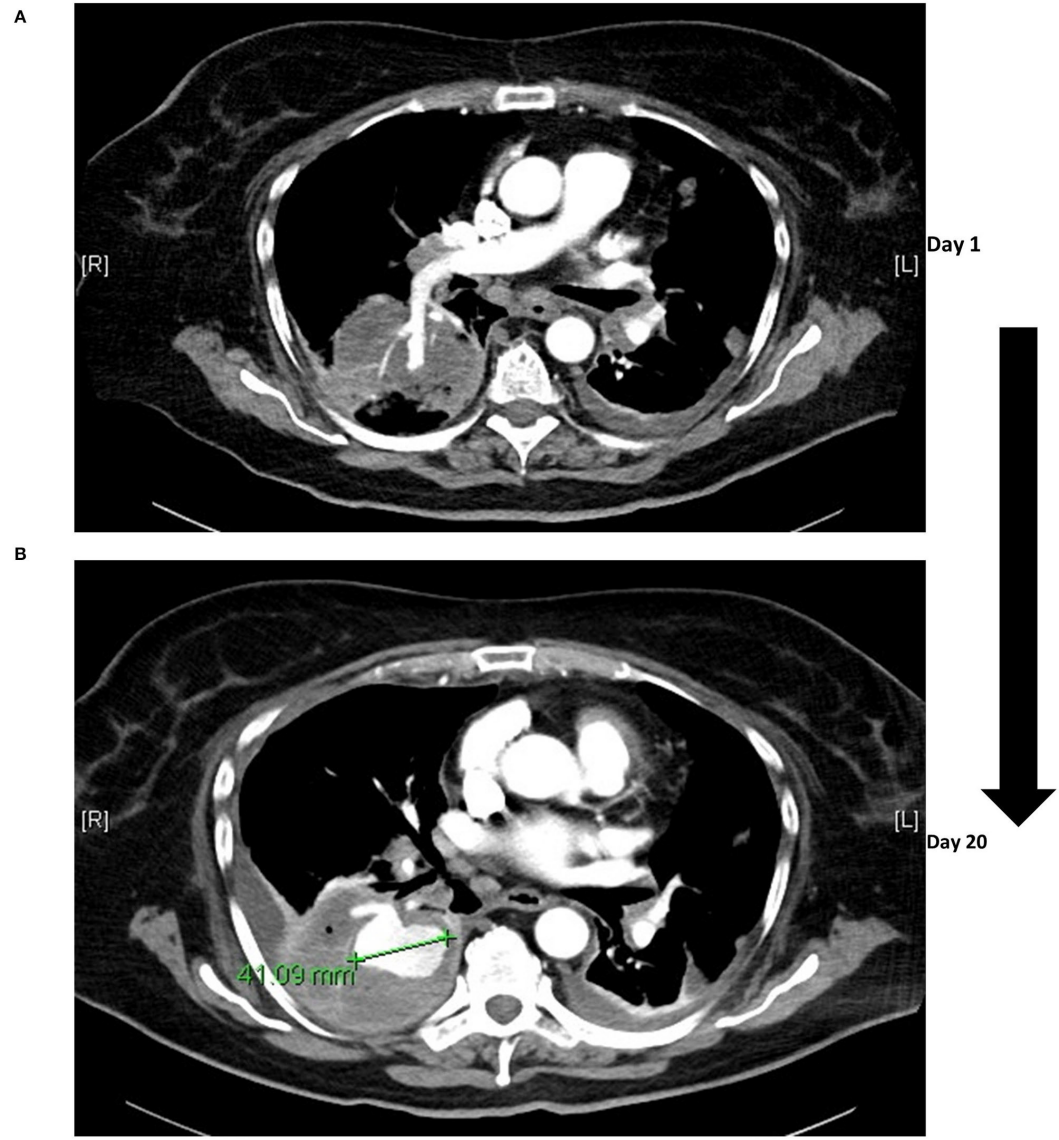
Infectious aneurysm of the pulmonary artery. The initial computed tomography was performed on the day of admission **(A)** with solely consolidation in the right basal lobe and an accentuated right lower lobe pulmonary artery. Over the course of 20 days an infectious pulmonary arterial aneurysm manifested with a diameter >4 cm **(B)**, coinciding with clinical signs of hemoptysis.

A 63-year-old Caucasian female was admitted to the intermediate care unit with a first diagnosis of diabetes mellitus and an infection-associated metabolic (keto)-acidosis (pH 7.16). Initially, the patient was hypovolemic, disoriented, tachypneic, hypoxemic, complained about epigastric pains, and loss of vision on the left eye. The serum glucose and the inflammation markers were markedly elevated (Table 1). The initial management included fluid resuscitation, insulin therapy and an empiric antibiotic treatment with piperacillin/tazobactam after blood- and urine-culture sampling. Due to persisting hypoxemia high-flow oxygen supplementation therapy was necessary and a CT was performed. This revealed signs of pyelonephritis, a renal vein thrombosis and bilateral subsegmental lung emboli co-localizing with multiple round and wedge-shaped infiltrates and a large consolidation in the right lower lobe (Figures 1, 2).

**Table 1:** Blood parameters at admission.

**pH (ven.)**	7.16
**pCO2 (ven.)**	23 mmHg
**HCO3- (ven.)**	8 mmol/L
**Base Excess**	−19.8 mmol/L
**Serum-Glucose**	33 mmol/L
**Serum-Hba1c**	14.2%
**Plasma-ketone bodies**	557.8 μmol/L
**C-Peptide**	3.28 ng/ml
**Insulin**	6 mU/ml
**Anti-GADA**	Negative
**Anti-IA2**	Negative
**Anti-Insulin**	Negative
**Serum-CRP**	387.7 mg/L
**Serum-PCT**	3.9 μg/L
**BSS 1 h**	77 mm
**BSS 2 h**	81mm
**WBC**	27,500/μl
**Neutrophils**	87.5%
**Eosinophils**	0%
**Basophils**	0.3%
**Monocytes**	4.9%
**Lymphocytes**	4.9%
**Urin-pH**	5.0
**Urin-Glucose**	+++
**Urin-Ketonbodies**	+++
**Anti-neutrophilicantibodies**	1:80 speckled
***KlebsiellaPneumoniae***	
**Time to positivity = 0 d,11 h,15 min** **Antibiogram** **[R= resistant,** **I= intermediate,** **S= sensible]** **Minimal inhibitory concentration (MIC)**	Ampicillin [R] ≤ 2.0 Ampicillin-Sulbactam [S] ≤ 2.0 Piperacillin-Tazobactam [S] ≤ 4.0 Cefuroxim [I] ≤ 1.0 Cefuroxim-Axetil [S] ≤ 1.0 Cefpodoxim [S] ≤ 0.25 Ceftriaxon [S] Cefotaxim [S] ≤ 1.0 Ceftazidim [S] ≤ 1.0 Gentamicin [S] ≤ 1.0 Levofloxacin [S] Ciprofloxacin [S ≤ 0.25] Moxifloxacin [S] ≤ 0.25 Meropenem [S] ≤ 0.25 Ertapenem [S] ≤ 0.5 Cotrimoxazol [S] ≤ 20.0

*With Hba1c, hemoglobin A1c, Anti-GADA, glutamic acid decarboxylase antibodies, Anti-IA2S, tyrosine phosphatase antibodies, CRP, C-reactive protein, PCT, Procalcitonin, BSS, Blood sedimentation speed, WBC, White blood cell count, ENA, Extractable nuclear antigen and ANCA, Anti-neutrophil cytoplasmatic antibody. KP species in urine and blood culture were determined via mass spectrometry under employment of a matrix assisted laser desorption ionization time-of-flight (MALDI-TOF) system (VITEK^®^ MS, bioMérieux Inc., Nürtingen, Germany) and comparison with the companies database (Myla^®^, bioMérieux Inc., Nürtingen, Germany). Minimal inhibitory concentration was assessed with VITEK^®^ 2 (bioMérieux Inc., Nürtingen, Germany) following manufacturer's instructions*.

As the majority of the multifocal nodules either depicted an “afferent-vessel sign”, characterized by close topographic proximity to the arterial flow area, septic pulmonary embolism (SPE) was suspected. A duplex sonography revealed a marginal circumferential thrombosis of the right common femoral vein, and a therapeutic anticoagulation regimen with tinzaparin (16,000IE) was initiated.

During ophthalmologic examination, the left amaurotic eye presented markedly swollen, while cranial magnet-resonance imaging with orbital cross-sectional imaging detected a choroidal effusion in the left bulbus, leading to the diagnosis of endogenous endophthalmitis.

**Figure 3:**
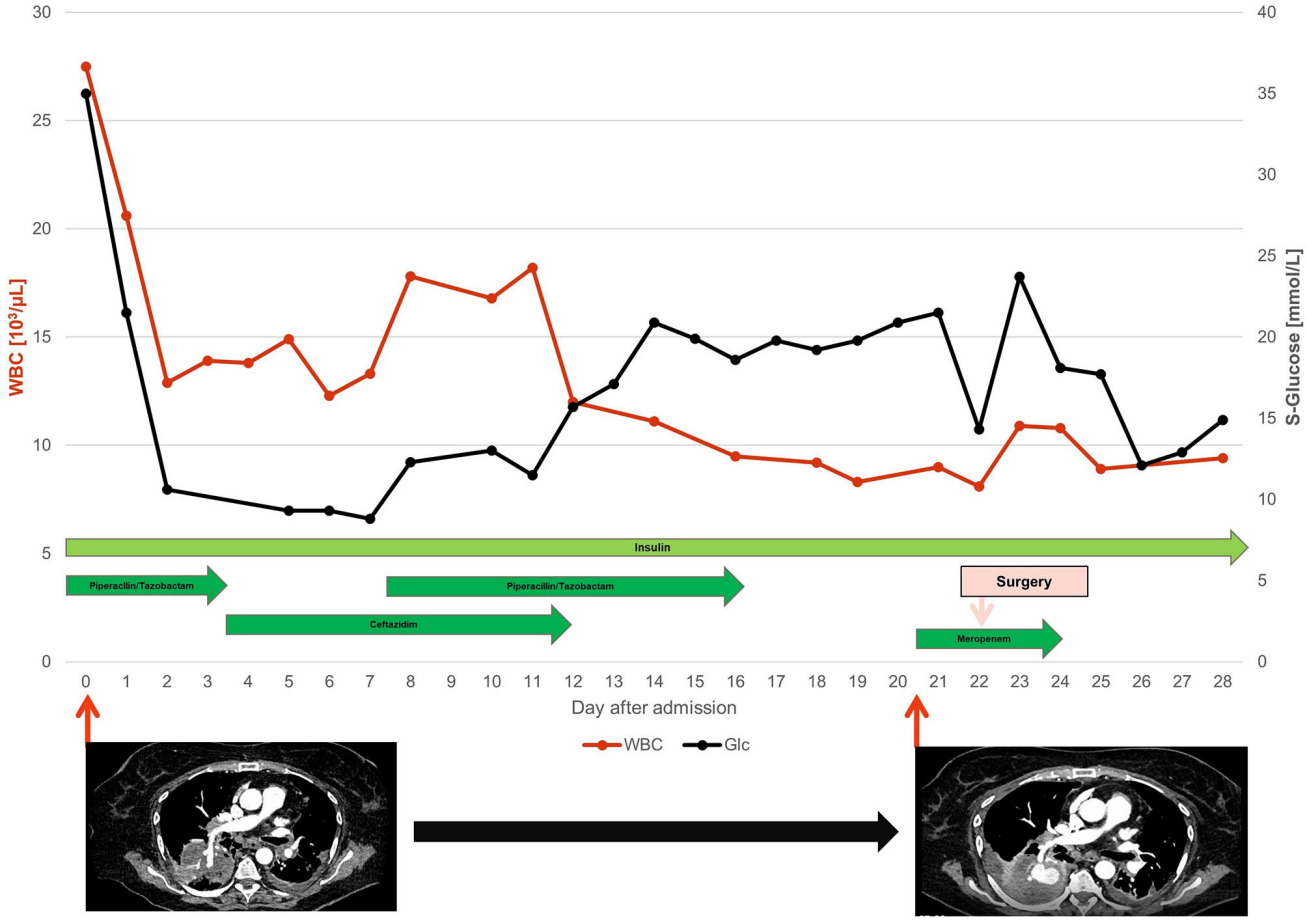
Overview on the clinical course and therapeutic management. With WBC, White blood cell count (red line) and Glc, Serum Glucose (black line) assessed by point of care testing. Duration of the applied antibiotic regimen is indicated by arrow length. Computer tomographic angiography of the thorax was performed twice, revealing progressive intrapulmonary infection of the right pulmonary subsegmental artery with manifestation of an infectious aneurysm (compare Figure 2).

**Figure 4:**
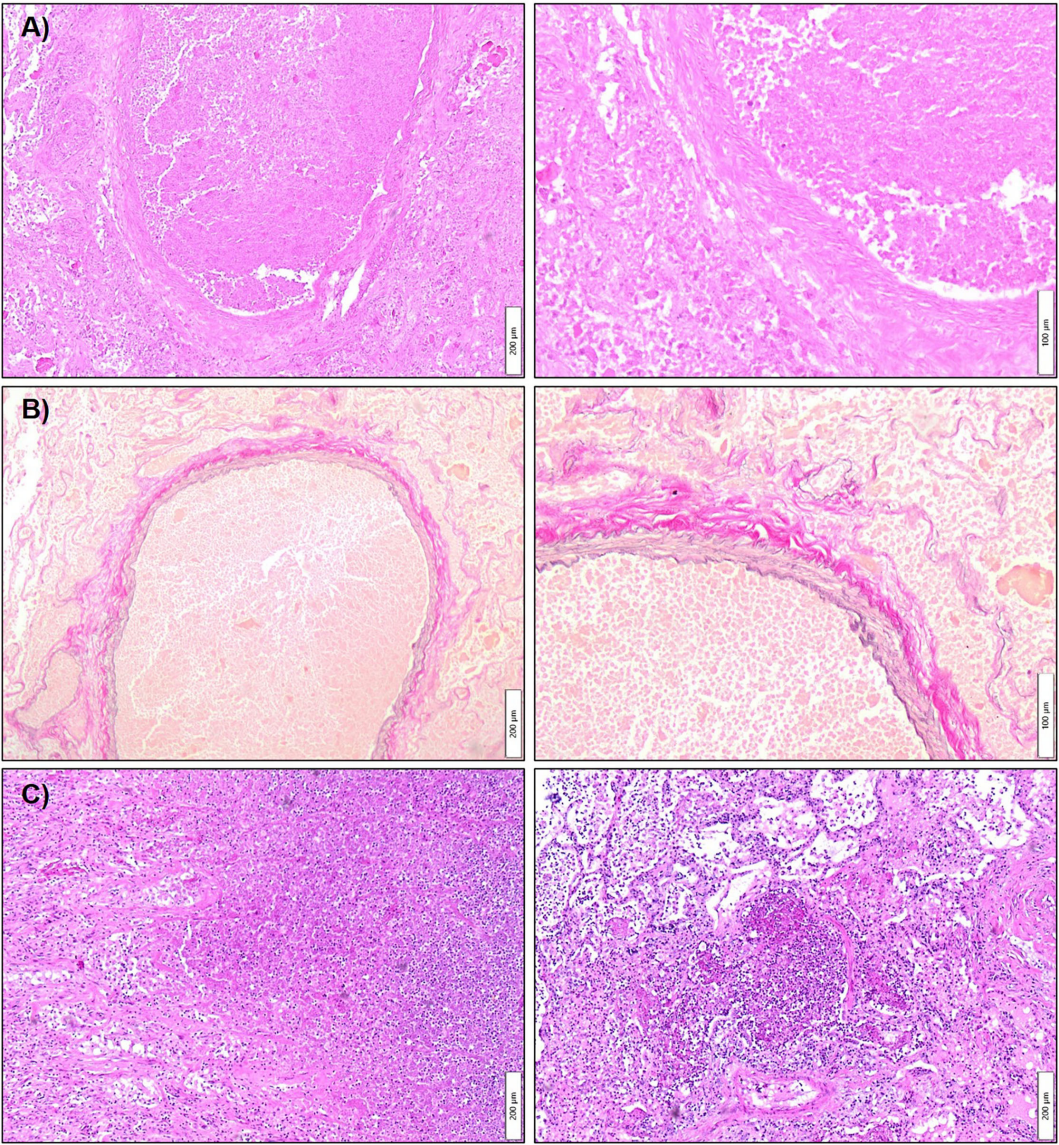
Histopathology of the infectious aneurysm of the inferior lobe pulmonary artery and concomitant necrotizing bronchopneumonia. Tissue sections were acquired from the resected right lower lobe comprising the infectious aneurysm. Histopathological work-up was performed with Hematoxylin-Eosin **(A,C)** and Elastica van Gieson **(B)** staining. In **(A)** depicted two magnifications show a dilated pulmonary artery branch occluded by a granulocyte rich fibrin thrombus with accompanying vascular wall necrosis. **(B)** Highlights the same area in a trichrome staining revealing the dual elastic fiber network. **(C)** Shows the partially necrotizing, fibrinous-purulent pneumonia adjacent to the vascular changes. Meanwhile, no signs of malignancy were noted in the investigated specimen.

Blood and urine culture tested positive for pan sensible *KP* (Table 1), and a diagnosis of pyelonephritis with consecutive urosepsis was made. The anti-infective regimen was subsequently de-escalated to ceftazidime in accordance with the tested antibiogram (Table 1). Secondary septic pulmonary and ocular embolisms associated with the *KP* sepsis were hypothesized. Following targeted therapy, the patient's vigilance and oxygenation status markedly improved, but glycemic control remained challenging even under a continuously intensified insulin regimen (Figure 3). For further diagnostic workup regarding SPE, transesophageal echocardiography was performed, which ruled out infectious endocarditis. However, the patient developed massive pulmonary hemorrhage, requiring repetitive inhalation with nebulized adrenaline. To elucidate the source of bleeding, a second chest CT was performed that showed an unexpected massive aneurysm of the right pulmonary artery (4 cm diameter). At this point, blood cultures were tested negative and inflammation markers had dropped substantially. Diagnosis of an infectious aneurysm secondary to the initial infection was assumed (Figures 2, 3). Upon diagnosis, antibiotic treatment was switched to meropenem (Figure 3), to empirically cover pathogenic resistance mechanisms, that may have manifested under the prolonged prior antibiotic regimen, in order to prevent pathogen related aneurysmal rupture. Surgical treatment as minimal invasive video-assisted thoracoscopic lobectomy of the right lower lobe including the infectious aneurysm was performed. Histopathological work-up depicted neither signs of malignancy of autoimmune disease nor direct pathogen detection. However, necrotizing and fibrinous purulent, partly gangrenous thromboembolic inflammation was noted (Figure 4). The patient made an uneventful recovery from the surgical procedure. On day 31 after admission the patient was respiratory stable, exhibited no reminiscent clinical or laboratory signs of infection and was discharged to a diabetic rehabilitation center.

## Discussion

Within the rare occurrences of infectious aneurysms, involvement of the pulmonary arteries is even more rarely seen (Papaioannou et al., 2014; Benhassen et al., 2018), while predictors for manifestation of infectious aneurysms following septic pulmonary embolism are absent. To date, only few cases of this disease have been described, and the reported fatality rate is higher than 50% (Bartter et al., 1988), especially if pulmonary hemorrhage occurs (Papaioannou et al., 2014). Furthermore, Gram-negative infections (like in the case of *KP* sepsis) were associated with an increased risk for rupture of the aneurysmatic wall (Jarrett et al., 1975).

*Klebsiella pneumoniae* is a member of the *Enterobacteriaceae* family and is a common pathogen in lower urinary tract, soft tissue and pulmonary infections. Thereby, pulmonary pathogenicity of virulent *KP* species has been recently emphasized by increasing isolation frequencies in respiratory samples over the past decade (Mendez et al., 2020; Braeken et al., 2021). Typically, if rarely, hyper virulent, hypermucoviscous *KP* species have been interlinked with systemic infection and sequential involvement of multiple organ sites (Fang et al., 2004; Chang et al., 2021), which is likely to have occurred in our patient despite absent genetic in depth characterization of the detected *KP* isolate. Indeed, pulmonary manifestation of systemic *KP* infection or more particular septic pulmonary embolism seems uncommon (Chang et al., 2021).

In 2005 Cook and colleagues defined the occurrence of septic pulmonary embolism by the presence of four criteria: (1) uni- or multifocal pulmonary infiltrates with (2) an active extra pulmonary infection site acting as embolic source under (3) exclusion of any malignant or non-malignant differential diagnosis. Finally, resolution of the pulmonary infiltrate under adequate antibiotic treatment is the 4th mandatory feature before the diagnosis can be secured (Cook et al., 2005).

Thus, firstly multiple bilateral nodular opacities or wedge-shaped infiltrates in close proximity to the pulmonary arterial circulation, depicting the SPE-suggestive “feeding vessel sign”, were present on the patient's chest CT scan (Lee et al., 2007; Ye et al., 2014; Chang et al., 2016). Although *KP* related SPE presents with a broad spectrum of radiologic signs including nodules, cavities, ground glass opacities or pleural effusions, the “feeding vessel sign” [11/14 patients, (Chou et al., 2015)] and wedge-shaped peripheral opacities [12/33, (Zhang et al., 2021)] were most frequently noticed in two distinct retrospective case-series.

Secondly, a pyelonephritis that led to urosepsis was identified as the extrapulmonary infection site. *KP* related lower urinary tract infections are rather frequent events (Paczosa and Mecsas, 2016; Shakya et al., 2017), while permissive hyperglycemia and glucosuria as present in our patients, have been reported as main risk factors(Lee et al., 2016). Additionally, secondary endogenous endophthalmitis is often seen especially in patients of Asian descent with systemic *KP* infection (Yang et al., 2007; Hagiya et al., 2013; Chang et al., 2016). SPE as pulmonary complication in *KP* infection is rarely described in Western patients (Cook et al., 2005). However, a systematic review analyzing case reports and case series of patients with SPE, applying the *Cook* criteria, in patients >14 years of age between 1979 and 2012 in a global perspective, refers to *KP* as the causative pathogen in 7.3% (11/151 patients) of the reviewed cases (Ye et al., 2014). Meanwhile, Yang and colleagues found diabetes mellitus to significantly increase the risk for septic metastases in a head-to-head comparison with a non-diabetic control group (Yang et al., 2007). Several case reports have been published wherein *KP* related SPE manifested in patients with diabetic metabolic decompensation (Zenda et al., 1995; Chang et al., 2015; Ojeda Gómez et al., 2019). A prospective single center cohort study performed by Lee and co-workers describes a significantly increased risk of *KP*-related SPE in diabetic patients with a Hba1c>9% [*OR* 5.66, 95%*CI* (2.01, 15.9)] (Lee et al., 2016). Furthermore, the authors infer that the hyperglycemia induced pathogenicity of KP is related to overexpression of critical genes for the synthesis and assembly of bacterial polysaccharide capsule. Also, neutrophilic phagocytotic activity against *KP* was compromised *in vitro* following incubation of *KP* with glucose prior to exposition to patient derived neutrophilic granulocytes (Lee et al., 2016). Referring these findings back to the presented case, our patient showed typical septic manifestation sites of *KP* related infectious metastatic disease. Moreover, the pronounced hyperglycemia in our patient is likely to have served as a major risk factor for septic dissemination (Rahimian et al., 2004; Keller et al., 2013).

Thirdly, possible differential diagnoses were ruled out by histopathology having shown no signs of malignancy or granulomatous lung disease.

Fourthly, antibiotic treatment led to a significant improvement of the clinical course, especially in terms of arterial oxygenation and lung function. On the contrary, iterated CT scanning showed no resolution of the initially observed bilateral infiltrates, as would be expected in the case of adequately treated SPE. Instead, our patient developed an aneurysm under anti-infective treatment in only a time period spanning 20 days (Figure 3). Of note, the patient had severe issues in terms of glycemic control in this time period, while diabetes was associated with the adverse outcome and death in patients with infective aortic aneurysms undergoing open surgery (Lau et al., 2015; Sörelius et al., 2019). Thereby, infectious aneurysms are believed to result either from septic thromboembolic occlusion of the pulmonary *vasa vasorum* of the pulmonary arteries from infective endocarditis or distinct septic thrombotic foci with concomitant invasive infection of the arterial wall. Thus, continuous recruitment of neutrophilic granulocytes and release of various proteases leads to progressive thinning of the arterial wall with resultant wall ectasia accompanied with a high risk of hemorrhage (Baddour et al., 2015; Habib et al., 2015; Majeed and Ahmad, 2021). A recent review on published cases with infectious aneurysms of pulmonary arteries between 1949 and 2018 described endocarditis and congenital heart defects as the main predisposing factors in almost half of the cases together (Benhassen et al., 2018). *Staphylococci or streptococci* were most frequently isolated, but also Gram-negative bacteria and various fungi have been occasionally reported (Jarrett et al., 1975; Müller et al., 2001; Ebisawa et al., 2018). However, no signs of endocarditis were present in our patient, while pulmonary infectious aneurysm have also been repetitively reported independent of present infectious endocarditis (Dransfield and Johnson, 2003; Benhassen et al., 2018; Alquichire-Luna et al., 2021).

To the best of our knowledge, only a single case-report on a *KP*-related infectious aneurysm has been reported by Alquichire-Luna and colleagues in a neutropenic patient with a history of acute myeloid leukemia. In contrast, our patient showed no state of immunosuppression besides decompensated type 2 diabetes. While the *KP* isolate in the referred case exhibited multiple antibiotic resistances (meropenem, piperacillin-tazobactam) predisposing an adverse clinical course, only pan sensible *KP* was identified in urine and blood (Table 1) from our patient. However, a recent meta-analysis of infection in patients with manifest type 2 diabetes found an increased risk of antibiotic-resistant infection with predominant involvement of the urinary tract [*OR* = 2.42, 95% *CI* = (1.83, 3.20)] and the lung [*OR* = 2.35, 95% *CI* = (1.49, 3.69)] (Carrillo-Larco et al., 2022), also predisposing these patients to an adverse clinical course. In our case, secondary formation of a subsegmental pulmonary arterial aneurysm was noted, which occurred most likely due to persistent sterile local inflammatory processes of the arterial wall. Conversely, indicators of systemic infection were tested negative after antibiotic treatment. Unfortunately, no general treatment recommendations on infectious aneurysms are present throughout the literature and in particular clear guidance on selection of the adequate antibiotic agent and treatment duration is missing (Baddour et al., 2015; Habib et al., 2015; Sörelius et al., 2020). Thus, the question after a superior preemptive antibiotic regime than the one administered in the case presented remains unanswered. Data only exists from a nationwide retrospective study from Sweden on treatment of infectious abdominal aortic aneurysms which suggested significant favorability in terms of postoperative mortality at 3 and 5 month of a post-operative antibiotic treatment regimen > 6 month in a multivariate analysis (Sörelius et al., 2016). However, the applicability of this study for the case presented remains questionable, as patients were treated either with endovascular or open surgical vascular repair including implantation of foreign body material into pre-infected tissue. Likewise, favorability of either microsurgical resection (current case) or interventional management (Alquichire-Luna et al., 2021) of manifested aneurysms in the pulmonary vasculature remains elusive and may rely on the referring center.

The present case-report underscores the dynamic character of local SPE related infection bearing the perilous potential to evolve to an infectious aneurysm. Comorbid diabetes mellitus might serve asan additional risk factor setting the stage for persistent *KP* infection under antibiotic treatment. Thus, underlying diabetes was present in 62% of patients with *KP* related liver abscess and subsequent extra hepatic metastatic disease (i.e., pulmonary and ophthalmic involvement) (Han, 1995) and in 68% of patients with *KP*-related endophthalmitis in a retrospective case series (Yang et al., 2007). Therefore, careful diagnostic work-up is necessary in patients presenting with hemoptysis and persistent signs of systemic infection including CT based angiography. Accordingly, central pulmonary aneurysms can be easily misinterpreted as hilar masses on native imaging techniques, seducing the pulmonologists to an invasive biopsy approach with potential fatal outcome (Dransfield and Johnson, 2003).

## Conclusion

*Klebsiella pneumoniae* may play a significant role in septic pulmonary embolic disease. However, metabolic decompensation of underlying diabetes mellitus, as often seen during infectious diseases, may serve as breeding ground for *KP* related septic embolisms including pulmonary involvement. CT based angiography should be performed quickly and prior to other invasive diagnostic procedures for avoiding iatrogenic aneurysmal rupture. However, identification of patients at risk for the development of aneurysmal disease resembles a major obstacle, as simple absence of current bacteremia not necessarily rules out persistent infectious or inflammatory reactions in already metastatically involved organs. Meanwhile, the overall manifestation rate following infectious endocarditis or simple bacteremia remains low. Hence, prospective studies to establish standardized guidelines are highly warranted for securing an early diagnosis and adequate treatment of the affected patients. However, such desirable tools are currently missing in the diagnostic quiver, which is why clinicians should be aware of pulmonary sequelae in patients with prolonged infectious disease with or without hemoptysis. The immunosuppressive capacity of permissive diabetic metabolic decompensation may serve as a perilous pro-infectious catalyzer that should not be underestimated.

## Data Availability Statement

The raw data supporting the conclusions of this article will be made available by the authors, without undue reservation.

## Ethics Statement

Ethical review and approval was not required for the study on human participants in accordance with the local legislation and institutional requirements. Written informed consent for participation was not required for this study in accordance with the national legislation and the institutional requirements.

## Author Contributions

JR, BF, and B-AB had the initial idea to perform this study. JR, BF, LH, and B-AB collected the samples and performed the clinical and radiological investigations. FL performed the histopathological analysis. JR, BF, and B-AB wrote the manuscript that was read and approved by all authors.

## Conflict of Interest

The authors declare that the research was conducted in the absence of any commercial or financial relationships that could be construed as a potential conflict of interest.

## Publisher's Note

All claims expressed in this article are solely those of the authors and do not necessarily represent those of their affiliated organizations, or those of the publisher, the editors and the reviewers. Any product that may be evaluated in this article, or claim that may be made by its manufacturer, is not guaranteed or endorsed by the publisher.

## References

[B1] Alquichire-Luna C. A. García-Bohórquez D. F. Hernández-Vargas J. C. García-Bohórquez J. A. Fajardo-Rivero J. E. (2021). Mycotic Pulmonary Aneurysm Managed With Covered Stent. Vasc. Endovascular Surg. 56:117–120. 10.1177/15385744211042179 34625008

[B2] Baddour L. M. Wilson W. R. Bayer A. S. FowlerJr V. G. Tleyjeh I. M. Rybak M. J.. (2015). Infective endocarditis in adults: diagnosis, antimicrobial therapy, and management of complications. Circulation. 132, 1435–1486. 10.1161/CIR.0000000000000296 26373316

[B3] Bartter T. Irwin R. S. Nash G. (1988). Aneurysms of the pulmonary arteries. Chest. 94, 1065–1075. 10.1378/chest.94.5.1065 3053058

[B4] Benhassen L. Højsgaard A. Allan Terp K. de Paoli F. (2018). Surgical approach to a mycotic aneurysm of the pulmonary artery presenting with hemoptysis—a case report and a review of the literature. Int. J. Surg. Case Rep. 50, 92–96. 10.1016/j.ijscr.2018.07.029 30092541 PMC6086216

[B5] Braeken D. C. W. Essig A. Panning M. Hoerster R. Nawrocki M. Dalhoff K.. (2021). Shift in bacterial etiology from the CAPNETZ cohort in patients with community-acquired pneumonia: data over more than a decade. Infection. 49, 533–537. 10.1007/s15010-021-01605-w 33774804 PMC8159805

[B6] Brown S. L. Busuttil R. W. Baker J. D. Machleder H. I. Moore W. S. Barker W. F. (1984). Bacteriologic and surgical determinants of survival in patients with mycotic aneurysms. J. Vasc. Surg. 1, 541–547. 10.1016/0741-5214(84)90040-5 6436514

[B7] Carrillo-Larco R. M. Anza-Ramírez C. Saal-Zapata G. Villarreal-Zegarra D. Zafra-Tanaka J. H. Ugarte-Gil C.. (2022). Type 2 diabetes mellitus and antibiotic-resistant infections: a systematic review and meta-analysis. J. Epidemiol. Community Health. 76, 75–84. 10.1136/jech-2020-216029 34326183 PMC8666814

[B8] Chang D. Sharma L. Dela Cruz C. S. Zhang D. (2021). Clinical epidemiology, risk factors, and control strategies of *Klebsiella pneumoniae* Infection. Front. Microbiol. 12, 750662. 10.3389/fmicb.2021.750662 34992583 PMC8724557

[B9] Chang Z. Gong Z. Zheng J. Ma Y. Liu Z. (2016). Computed tomography features of septic pulmonary embolism caused by *Klebsiella pneumoniae* liver abscess associated with extrapulmonary metastatic infection. J. Comput. Assist. Tomogr. 40, 364–369. 10.1097/RCT.0000000000000383 26938693

[B10] Chang Z. Zheng J. Ma Y. Liu Z. (2015). Analysis of clinical and CT characteristics of patients with *Klebsiella pneumoniae* liver abscesses: an insight into risk factors of metastatic infection. Int. J. Infect. Dis. 33, e50–e54. 10.1016/j.ijid.2014.12.041 25555624

[B11] Chou D. W. Wu S. L. Chung K. M. Han S. C. (2015). Septic pulmonary embolism caused by a *Klebsiella pneumoniae* liver abscess: clinical characteristics, imaging findings, and clinical courses. Clinics (Saõ Paulo). 70, 400–407. 10.6061/clinics/2015(06)03 26106957 PMC4462570

[B12] Cook R. J. Ashton R. Aughenbaugh G. Ryu J. (2005). Septic pulmonary embolism: presenting features and clinical course of 14 patients. Chest. 128, 162–166. 10.1378/chest.128.1.162 16002930

[B13] Dransfield M. T. Johnson J. E. (2003). A Mycotic Pulmonary Artery Aneurysm Presenting as an Endobronchial Mass. Chest. 124, 1610–1612. 10.1378/chest.124.4.1610 14555599

[B14] Ebisawa K. F. Nishimura S. Yamamoto S. Ohji G. Iwata K. (2018). Mycotic aneurysm caused by Edwardsiella tarda successfully treated with stenting and suppressive antibiotic therapy: a case report and systematic review. Ann. Clin. Microbiol. Antimicrob. 17. 10.1186/s12941-018-0273-x 29747632 PMC5944098

[B15] Fang C. T. Chuang Y. P. Shun C. T. Chang S. C. Wang J. T. (2004). A novel virulence gene in *Klebsiella pneumoniae* strains causing primary liver abscess and septic metastatic complications. J. Exp. Med. 199, 697–705. 10.1084/jem.20030857 14993253 PMC2213305

[B16] Habib G. Lancellotti P. Antunes M. J. Bongiorni M. G. Casalta J. P. Del Zotti F.. (2015). 2015 ESC Guidelines for the management of infective endocarditis. Eur. Heart J. 36, 3075–3123. 10.1093/eurheartj/ehv319 26320109

[B17] Hagiya H. Kuroe Y. Nojima H. Otani S. Sugiyama J. Naito H.. (2013). Emphysematous liver abscesses complicated by septic pulmonary emboli in patients with diabetes: two cases. Intern. Med. 52, 141–145. 10.2169/internalmedicine.52.8737 23291690

[B18] Han S.-H. (1995). Review of hepatic abscess from Klebsiella pneumoniae—an association with diabetes mellitus and septic endophthalmitis. West. J. Med. 162, 220–224. 7725704 PMC1022703

[B19] Jarrett F. Darling R. C. Mundth E. D. Austen W. G. (1975). Experience With Infected Aneurysms of the Abdominal Aorta. Arch. Surg. 110, 1281–1286. 10.1001/archsurg.1975.01360170021002 1191021

[B20] Keller J. J. Tsai M. C. Lin C. C. Lin Y. C. Lin H. C. (2013). Risk of infections subsequent to pyogenic liver abscess: a nationwide population-based study. Clin. Microbiol. Infect. 19, 717–722. 10.1111/1469-0691.12027 23034092

[B21] Lau C. Gaudino M. De Biasi A. R. Munjal M. Girardi L. N. (2015). Outcomes of open repair of mycotic descending thoracic and thoracoabdominal aortic aneurysms. Ann. Thorac. Surg. 100, 1712–1717. 10.1016/j.athoracsur.2015.05.067 26277557

[B22] Lee C. H. Chen I. L. Chuah S. K. Tai W. C. Chang C. C. Chen F. J.. (2016). Impact of glycemic control on capsular polysaccharide biosynthesis and opsonophagocytosis of *Klebsiella pneumoniae*: implications for invasive syndrome in patients with diabetes mellitus. Virulence 7, 770–778. 10.1080/21505594.2016.1186315 27159655 PMC5029304

[B23] Lee S. J. Cha S. I. Kim C. H. Park J. Y. Jung T. H. Jeon K. N.. (2007). Septic pulmonary embolism in Korea: Microbiology, clinicoradiologic features, and treatment outcome. J. Infect. 54, 230–234. 10.1016/j.jinf.2006.04.008 16750858

[B24] Macbeth G. A. Rubin J. R. McIntyre K. E. Goldstone J. Malone J. M. (1984). The relevance of arterial wall microbiology to the treatment of prosthetic graft infections: graft infection vs. arterial infection. J. Vasc. Surg. 1, 750–756. 10.1016/0741-5214(84)90005-3 6436515

[B25] Majeed H. Ahmad F. (2021). Mycotic Aneurysm. StatPearls. Available at: https://www-1ncbi-1nlm-1nih-1gov-1v67xgbt405eb.han.mh-hannover.de/books/NBK560736/32809571

[B26] Mendez L. Pedrosa A. Caneiras C. (2020). Growing importance of gram-negative isolates in respiratory samples. Eur. Respir. J. 56, 2027. 10.1183/13993003.congress-2020.2027

[B27] Müller B. T. Wegener O. R. Grabitz K. Pillny M. Thomas L. Sandmann W. (2001). Mycotic aneurysms of the thoracic and abdominal aorta and iliac arteries: Experience with anatomic and extra-anatomic repair in 33 cases. J. Vasc. Surg. 33, 106–113. 10.1067/mva.2001.110356 11137930

[B28] Oderich G. S. Panneton J. M. Bower T. C. Cherry K. J. Rowland C. M. Noel A. A.. (2001). Infected aortic aneurysms: aggressive presentation, complicated early outcome, but durable results. J. Vasc. Surg. 34, 900–908. 10.1067/mva.2001.118084 11700493

[B29] Ojeda Gómez J. S. A. Carrillo Bayona J. A. Morales Cifuentes L. C. (2019). Septic pulmonary embolism secondary to *Klebsiella pneumoniae* epididymitis: case report and literature review. Case Rep. Radiol. 2019, 1–5. 10.1155/2019/5395090 31016062 PMC6444252

[B30] Osler W. (1885). The gulstonian lectures, on malignant endocarditis. Br. Med. J. 1, 467–470. 10.1136/bmj.1.1262.467 20751186 PMC2255866

[B31] Paczosa M. K. Mecsas J. (2016). Klebsiella pneumoniae: Going on the Offense with a Strong Defense. Microbiol. Mol. Biol. Rev. 80, 629. 10.1128/MMBR.00078-15 27307579 PMC4981674

[B32] Papaioannou V. Mikroulis D. Chrysafis I. Fotakis S. Pneumatikos I. (2014). Hemoptysis due to a mycotic pulmonary artery aneurysm in an injecting drug user. Thorac. Cardiovasc. Surg. 62, 453–455. 10.1055/s-0032-1330952 23250844

[B33] Qureshi T. Hawrych A. B. Hopkins N. F. G. (1999). Mycotic aneurysm after percutaneous transluminal femoral artery angioplasty. J. R. Soc. Med. 92, 255–256. 10.1177/014107689909200515 10472268 PMC1297183

[B34] Rahimian J. Wilson T. Oram V. Holzman R. S. (2004). Pyogenic liver abscess: recent trends in etiology and mortality. Clin. Infect. Dis. 39, 1654–1659. 10.1086/425616 15578367

[B35] Shakya P. Shrestha D. Maharjan E. Sharma V. K. Paudyal R. (2017). ESBL production among *E. coli and Klebsiella spp*. Causing urinary tract infection: a hospital based study. Open Microbiol. J. 11, 23–30. 10.2174/1874285801711010023 28553414 PMC5427687

[B36] Sörelius K. Budtz-Lilly J. Mani K. Wanhainen A. (2019). Systematic review of the management of mycotic aortic aneurysms. Eur. J. Vasc. Endovasc. Surg. 58, 426–435. 10.1016/j.ejvs.2019.05.004 31320247

[B37] Sörelius K. Wanhainen A. Furebring M. Björck M. Gillgren P. Mani K.. (2016). Nationwide study of the treatment of mycotic abdominal aortic aneurysms comparing open and endovascular repair. Circulation 134, 1822–1832. 10.1161/CIRCULATIONAHA.116.024021 27799273

[B38] Sörelius K. Wanhainen A. Mani K. (2020). Infective native aortic aneurysms: call for consensus on definition, terminology, diagnostic criteria, and reporting standards. Eur. J. Vasc. Endovasc. Surg. 59, 333–334. 10.1016/j.ejvs.2019.11.008 32131984

[B39] Yang C. S. Tsai H. Y. Sung C. S. Lin K. H. Lee F. L. Hsu W. M. (2007). Endogenous klebsiella endophthalmitis associated with pyogenic liver abscess. Ophthalmology 114. 10.1016/j.ophtha.2006.12.035 17467526

[B40] Ye R. Zhao L. Wang C. Wu X. Yan H. (2014). Clinical characteristics of septic pulmonary embolism in adults: a systematic review. Respir. Med. 108, 1–8. 10.1016/j.rmed.2013.10.012 24183289

[B41] Zenda T. Araki I. Hiraiwa Y. Miyayama S. Masunaga T. Takeda Y.. (1995). Septic pulmonary emboli secondary to pyogenic liver abscess in a diabetic patient. Intern. Med. 34, 42–45. 10.2169/internalmedicine.34.42 7718979

[B42] Zhang X. Yang Q. Gao B. Wang J. Tian L. Hua J.. (2021). *Klebsiella pneumoniae* infection associated septic pulmonary embolism in an emergency department from east China. Ann. Palliat. Med. 10, 1521–1529. 10.21037/apm-19-648 33183047

